# A discrete choice experiment analysis to understand patient preferences for multiple myeloma treatments

**DOI:** 10.3389/fonc.2025.1628121

**Published:** 2025-09-16

**Authors:** Beth Faiman, Hoa H. Le, Julie Laurent, Saurabh Patel, Agne Paner-Straseviciute, Xinke Zhang, Joseph Mikhael

**Affiliations:** ^1^ Cleveland Clinic, Cleveland, OH, United States; ^2^ Johnson & Johnson, Horsham, PA, United States; ^3^ Carenity, Paris, France; ^4^ Translational Genomics Research Institute, Phoenix, AZ, United States

**Keywords:** discrete choice experiment, multiple myeloma, patient preferences, trade-offs, treatment attributes

## Abstract

**Introduction:**

Multiple myeloma (MM) is a malignant plasma cell disorder characterized by the clonal expansion of abnormal plasma cells within the bone marrow. The management of relapsed/refractory multiple myeloma (RRMM) represents a significant challenge as the disease relapses or becomes refractory to previous treatments. Recent advances in therapy have expanded RRMM treatment options. This study aimed to gain a deeper understanding of patients' treatment preferences regarding available therapeutic options.

**Methods:**

This study was designed as a non-interventional descriptive cross-sectional study based on an online discrete choice experiment (DCE) among adult RRMM patients living in the between USA November 2023 and March 2024. The survey included attributes and levels derived from an extensive literature review and guided interviews conducted with MM patients. Preference data were analyzed using a conditional logistic (CL) regression model and relative attribute importance (RAI) scores were calculated. Patients’ willingness to trade off overall response rate (ORR) was evaluated using the partworth utilities estimated from the CL model.

**Results:**

149 MM patients completed the survey; 66% had received 1–2 prior lines of therapy, 15% three prior lines, 19% four or more prior lines. Patients significantly preferred treatments with longer progression-free survival (PFS) and overall survival (OS) and higher ORR (RAI: 36.4% and 22.1%, respectively). With respect to adverse events assessed in this study, patients expressed concern for cytokine release syndrome (CRS) (RAI: 15.2%) and infections (RAI: 11.9%). In contrast, nail/skin disorders, duration of hospitalization, and taste disorder were less important to patients. Patients would be willing to accept a high risk of CRS (72% over no risk) to gain 29% increase in ORR.

**Conclusions:**

Patients showed a clear preference for treatment efficacy (PFS/OS and ORR). This study confirmed patients’ valuation on treatment attributes in the new treatment landscape and highlighted the importance of shared treatment decision-making for optimal clinical outcomes.

## 1 Introduction

Multiple myeloma (MM) is a malignant plasma cell disorder characterized by the clonal expansion of abnormal plasma cells within the bone marrow (1). As the second most common hematologic malignancy, MM accounts for approximately 10% of all blood cancers, with approximately 36,000 new cases diagnosed in 2024 in the United States alone (2, 3). MM predominantly affects older adults, with a median age of diagnosis around 70 years, and has a slightly higher incidence in males than females (1, 4). The incidence is also twice as high in those of African descent and is diagnosed at a young age in African American and Latino American patients. This hematologic disease manifests through various debilitating symptoms due to the accumulation of malignant plasma cells, impacting normal blood cell production, renal function and bone integrity, resulting in fatigue, extensive bony pain, infections and the need for dialysis in certain patients. Consequently, MM places a substantial cost and quality-of-life (QoL) burden on patients and the healthcare system.

MM is a progressive, incurable disease characterized by cycles of remission and relapse, especially in advanced stages, which are known as relapsed refractory multiple myeloma (RRMM). RRMM is marked by either the reappearance of disease symptoms after prior improvement or resistance to existing therapies, with disease progression observed during or within 60 days post-treatment (5). Patients frequently undergo multiple lines of therapy, with each subsequent regimen demonstrating reduced efficacy and shorter duration of remission (6). Patients who have been exposed to proteasome inhibitors (PIs), monoclonal antibodies (mAbs) targeting CD38, immunomodulatory agents (IMiDs, triple-class exposed [TCE]), have particularly poor outcomes and need new treatment options with different mechanisms of action (3). The recent introduction of chimeric antigen receptor T-cell (CAR-T) therapy has dramatically improved outcomes in myeloma with deeper and more durable remissions than prior therapies, but also with challenges in accessing this complex therapy (7).

Recently, bispecific antibody therapies have emerged as promising options for RRMM patients. The FDA has approved several of these therapies, including teclistamab, elranatamab and talquetamab, for TCE patients who have undergone at least four prior lines of therapy. Teclistamab and elranatamab, both targeting B-cell maturation antigen (BCMA) and CD3 receptors, have also shown promising efficacy, with teclistamab achieving an ORR of 65% (8) and elranatamab demonstrating an ORR of 61% (9). Talquetamab, an IgG4 antibody, targets G protein-coupled receptor family C group 5 member D (GPRC5D) and CD3 receptors and facilitates T-cell–mediated lysis of MM-specific cells, with a reported overall response rate (ORR) of 67-74% (10). These recent advances illustrate the growing range of options available for the treatment of RRMM but also highlight the complexity of decision making for patients affected by this disease.

In parallel with the approval of bispecific antibodies, additional T-cell–redirecting therapies are under development, including trispecific antibodies that may offer enhanced efficacy by targeting multiple tumor antigens and reduce the risk of immune escape. For example, early clinical results for JNJ-5322, a next-generation trispecific antibody that simultaneously targets BCMA and GPRC5D while engaging CD3, were presented at the 2025 European Hematology Association (EHA) Congress and demonstrated encouraging anti-myeloma activity in heavily pretreated patients (11).

While prior studies have examined RRMM treatment preferences, few have explored trade-offs specific to T-cell redirection therapies, particularly regarding side effects such as CRS, infections, and taste or skin symptoms. As novel agents with distinct benefit-risk profiles continue to emerge, understanding how patients prioritize efficacy, safety, and treatment convenience is essential to support informed, personalized treatment decisions. In this context, it is increasingly important for clinicians to understand patient preferences to select therapies that align with patient values, improve adherence and minimize the risk of premature discontinuation.

Discrete choice experiments (DCEs) are particularly useful for capturing patient priorities and trade-offs by presenting respondents with hypothetical scenarios that reflect realistic treatment attributes. DCEs, which are widely used in health economics and patient-centered research, provide insight into factors such as efficacy, side-effect profiles, and treatment administration that influence the decision-making process, ultimately supporting clinicians in offering personalized care (12, 13).

In this study, we applied DCE methodology to explore the factors influencing treatment preferences among RRMM patients who have received at least one prior line of therapy. We aim to highlight the specific considerations and trade-offs that shape patient decision making in light of recent therapeutic developments.

## 2 Materials and methods

### 2.1 Study design and data sources

This descriptive, cross-sectional, observational, non-interventional, online stated preference survey was conducted in the United States of America (USA) from November 2023 to March 2024.

Data were collected through the Carenity patient community platform, local partnerships and online social media campaigns. The Carenity platform is an online patient community, launched in 2011, where patients affected by chronic disease can share their experiences, find health-related information, and contribute to medical research by participating in online studies. Partnerships were developed with local organizations (patient organizations or market research agencies) who invited their own communities/members to participate in the survey.

### 2.2 Ethical considerations

This study was conducted in accordance with the study protocol, the Declaration of Helsinki, the International Society of Pharmacoepidemiology guidelines for Good Pharmacoepidemiology Practices, and the General Data Protection Regulation. The protocol and survey materials were submitted for ethical review, and written approval was obtained from the Institutional Review Board (WIRB-Copernicus) in the USA in November 2023. All patients completed the informed consent form before any patient data were collected.

### 2.3 Study overview

Eligible patients were consenting adults (at least 18 years of age), living in the USA with a self-reported diagnosis of RRMM who had received at least one prior line of treatment for MM. Respondents who did not meet these criteria or had incomplete data (i.e., those who had never started or completed the questionnaire) were excluded.

The questionnaire consisted of 32 questions (**Appendix 1 in Supplementary Materials**), divided into four parts: A) Screener, B) DCE to elicit patients’ preferences, C) Sociodemographic and medical profile, D) Impact of the disease on life and treatment burden.

### 2.4 Attributes and level development

A literature review was conducted with the objective of identifying published clinical evidence on product characteristics that were previously considered to be key in patients’ treatment decisions. Attributes related to treatment efficacy were identified, with two main categories of study endpoints: those measuring the efficacy in terms of time elapsed before the occurrence of a particular event (e.g., overall survival [OS], progression-free survival [PFS], time to next treatment [TTNT], event-free survival [EFS], duration of response [DoR]) and endpoints relating to response to treatment, including the overall response rate (ORR), very good partial response (VGPR) rate and partial response (PR) rate. In the context of DCE, the efficacy attributes most commonly reported in the literature were OS, PFS and ORR. These were also among the most readily comprehensible to patients.

A substantial number of adverse events (AEs) associated with RRMM treatments have been reported in the literature. Consequently, a number of attributes related to the safety profile of the treatments were identified. The most commonly reported AEs were pain, neuropathy, infections, digestive disorders, anemia, cytokine release syndrome (CRS), vision disturbances and skin disorders. Given the increasing clinical use of bispecific therapies in RRMM patients, attributes such as CRS, infection and GPRC5D-related symptoms were prioritized to better assess patient preferences regarding their distinct safety profiles.

A further crucial aspect of a treatment is its mode of administration. The literature review identified several characteristics associated with treatment, including its frequency, its method of administration, localization of administration and necessity for monitoring.

The selection of levels was based on the clinical profiles of three bispecific antibody therapies approved in the US, namely talquetamab (14), elranatamab (15) and teclistamab (16), as well as conventional treatments based on a real-world study (17–21).

A qualitative study was conducted to ensure that the attributes were comprehensive and relevant. Six MM patients were interviewed to explore their perspectives on the most important factors when selecting a treatment for MM, as well as their expectations of MM treatments.

The final DCE design consisted of seven attributes, with the levels corresponding to each attribute described in **Table 1**.

**Table 1:** Attributes and levels included in the DCE models for MM patients.

Field	Attributes	Levels
Treatment efficacy	Time without progression of multiple myeloma and lifespan	- Time without progression: 4 months; lifespan: 9 months (reference) - Time without progression: 12 months; lifespan: 22 months - Time without progression: 15 months; lifespan: 22 months - Time without progression: 14 months; lifespan: 30 months
	Likelihood of responding to treatment	- 30 of 100 patients (reference) - 60 of 100 patients - 74 of 100 patients
Safety	Immune storm (CRS, Cytokine Release Syndrome)	- No risk: 0 of 100 patients (reference) - 56 of 100 patients, mild to moderate - 72 of 100 patients, mild to moderate
	Infections	- 50 of 100 patients; 13% severe, 37% mild (reference) - 67 of 100 patients; 32% severe, 35% mild - 76 of 100 patients; 45% severe, 31% mild
	Skin and/or nail disorders	- No risk: 0 of 100 patients (reference) - Skin disorders: 43 of 100 patients. - nail disorders: 68 of 100 patients
	Taste disorder	- No risk: 0 of 100 patients (reference) - 46 of 100 patients
Hospitalization at treatment initialization	Hospitalization to start treatment protocol	- No hospital days for monitoring (reference) - 5 days in hospital for monitoring - 10 days in hospital for monitoring

### 2.5 DCE design

**Figure 1:**
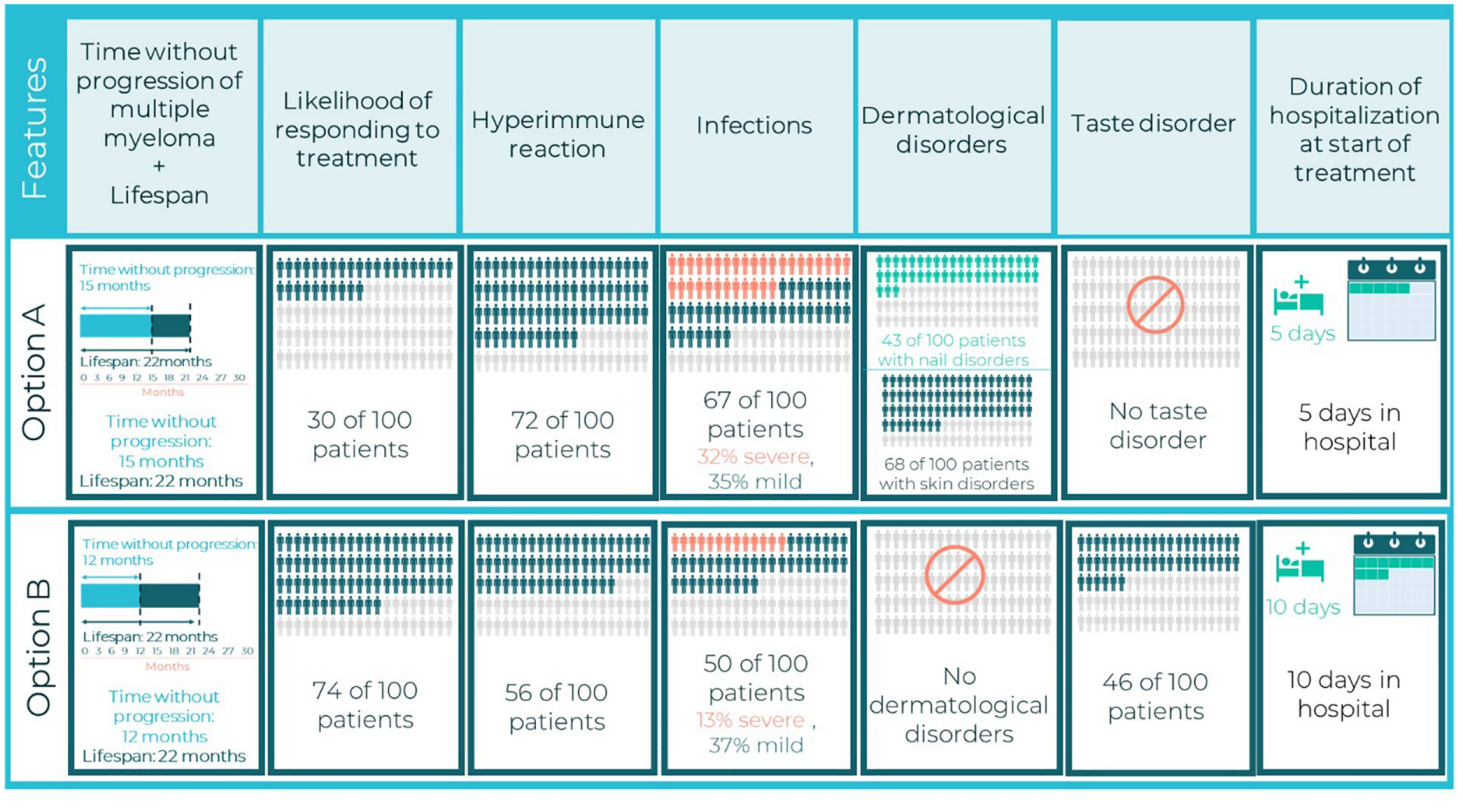
Example of DCE choice card.

The combinations of attribute levels displayed for each hypothetical treatment option, within each choice task in a DCE, were generated with a D-efficient experimental design to ensure that the choice tasks collected the maximum amount of information about the trade-offs between the attributes (12, 22, 23). The experimental design was generated using the *AlgDesign* library of the R software (version 1.2.1) (24). DCE design comprised 24 choice tasks, which were grouped into three blocks of eight tasks each. To minimize the cognitive burden of the DCE survey, patients were randomized to one of the three blocks. Across the choice tasks, patients were repeatedly asked to choose between two mutually exclusive hypothetical treatment alternatives (Treatment A or Treatment B) with different levels of benefits/risks and modes of administration (**Figure 1**). In addition to these eight experimental choice tasks, patients also completed one internal validity choice task. One choice task was repeated to assess whether patients were consistent in their choices (whether patients chose the same option as they had selected previously).

### 2.6 Statistical analysis

Based on the number of attributes, choice sets and alternatives, a sample size of 125 patients was deemed sufficient for DCE (25).

Descriptive statistics were used to examine the sociodemographic characteristics and all variables that were not directly related to the DCE methodology. Categorical variables were reported descriptively using frequencies and percentages, while continuous variables were presented as mean ± SD, median, lower and upper quartile, minimum and maximum values.

A conditional logistic (CL) regression model was used to analyze the patients’ treatment preferences. This model estimates the patients’ sensitivities to changes in the treatment attributes, also referred to as parthworth utilities, relative to a reference level. The estimated parthworth utilities were then used to calculate scores of relative attribute importance (RAI). RAI scores are conditional on the range of attribute levels, with a sum of 100%. They serve to illustrate the contribution of each attribute to treatment preferences. No covariates were incorporated into the initial CL model as there are no *a priori* variables that are known to influence the DCE results. The CL model was constructed to include all the treatment attributes described previously and presented in the DCE choice cards. These treatment attributes were coded as categorical variables. The reference levels were defined as the least favorable treatment characteristics (**Table 1**). To identify the preferred levels within a specific treatment attribute, comparisons were made between the CL coefficient (i.e., partworth utility) of the level of interest and the reference level’s partworth utility, which was constrained at 0 (26).

Based on the partworth utilities evaluated in the main CL model, the patients’ willingness to trade off for ORR and the overall utilities of each treatment option were calculated using the random utility model (27).

To understand potential differences in patient preferences, subgroup analyses were conducted by key variables of interest including number of prior lines of treatment, age, gender, disease duration, living area (urban, suburban, rural), income, activity level, and treatment history.

All tests were bidirectional and a p-value <0.05 was considered statistically significant. Data management and statistical analyses were conducted using R software version R 4.0.5.

## 3 Results

### 3.1 Patient demographics and clinical characteristics at study inclusion

**Figure 2:**
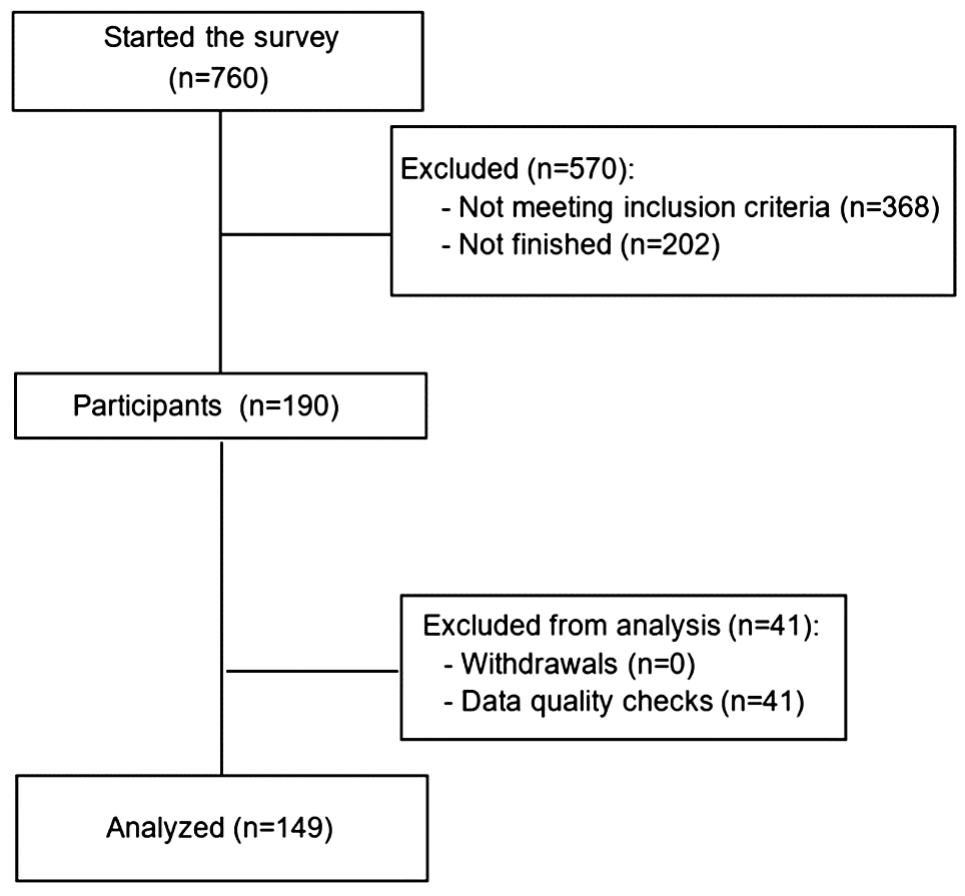
Flowchart of participants.

Overall, 149 patients with MM met the inclusion criteria and completed the survey (**Figure 2**). Patient sociodemographic and clinical characteristics are presented in **Table 2**. The mean age of patients was 63 years and 51% were women. Among them, 69% were White, 68% had a bachelor’s degree or higher and 46% lived in an urban area. The median disease duration was five years and 18.8% of patients had at least 4 prior lines of treatments. Most patients (93.3%) were receiving treatment for MM at the time of the survey, the most frequently received therapeutic agents being monoclonal antibodies (46.0%). A total of 12.9% of patients were undergoing CAR-T therapies, 12.9% BCMA-targeted bispecific antibody and 3.6% GPRC5D-targeted bispecific antibody. Overall, 58.4% of patients were exposed to triple-class therapy, and 30.2% had prior bispecific or CAR-T therapy.

**Table 2:** Patient sociodemographic characteristics.

Variable	Overall population (N=149)
Patient age, years	
Mean (SD)	62.5 (9.2)
Patient gender	
Male, n (%)	72 (48.3%)
Female, n (%)	76 (51.0%)
Other/Unknown, n (%)	1 (0.7%)
Patient races, ethnicities, or origins ^a^	
Hispanic, n (%)	12 (8.1%)
Black n (%)	30 (20.1%)
White, n (%)	103 (69.1%)
Other/Unknown, n (%)	7 (4.7%)
Highest educational or equivalent work-related qualification achieved	
2-year college degree and below, n (%)	48 (32.2%)
Bachelor's degree, n (%)	58 (38.9%)
Master's degree and above, n (%)	43 (28.9%)
Urbanization level of living area	
Urban area, n (%)	68 (45.6%)
Suburban area, n (%)	61 (40.9%)
Rural area, n (%)	20 (13.4%)
Relapse/remission status	
MM is currently relapsing/MM is refractory, n (%)	77 (51.7%)
MM is currently in remission, n (%)	72 (48.3%)
Disease duration, years	
Median (IQR)	5.0 (3.0-7.5)
Type of health insurance	
State/Public insurance, n (%)	49 (32.9%)
Private insurance, n (%)	55 (36.9%)
Combination of private and state/public insurance, n (%)	44 (29.5%)
I don't know, n (%)	1 (0.7%)
Number of other conditions outside of MM	
None, n (%)	41 (27.5%)
One, n (%)	41 (27.5%)
More than one, n (%)	67 (45.0%)
Current MM treatment status	
Currently receiving treatment for MM, n (%)	139 (93.3%)
Not currently receiving treatment for MM, n (%)	10 (6.7%)
Number of prior lines of treatment	n=149
1 prior line of treatment, n(%)	58 (38.9%)
2 prior lines of treatment, n(%)	40 (26.8%)
3 prior lines of treatment, n(%)	23 (15.4%)
4 prior lines of treatment, n(%)	16 (10.7%)
5 or more prior lines of treatment, n(%)	12 (8.1%)
Treatments currently received for MM ^a^	n=139
Selinexor, n (%)	19 (13.7%)
Proteasome inhibitors, n (%)	33 (23.7%)
Immunomodulatory drug, n (%)	59 (42.4%)
Monoclonal antibody anti-CD38, n (%)	64 (46.0%)
BCMA/CD3 Bispecific Ab, n (%)	18 (12.9%)
GPRC5D/CD3 Bispecific Ab, n (%)	5 (3.6%)
CAR-T cells, n (%)	18 (12.9%)
Stem cell transplant, n (%)	7 (5.0%)
Other, n (%) ^b^	12 (8.6%)
Triple-class therapy exposure ^c^	
Yes, n (%)	87 (58.4%)
No, n (%)	62 (41.6%)
Prior BCMA Bispecific Ab or CAR-T cells therapies ^d^	
Yes, n (%)	45 (30.2%)
No, n (%)	104 (69.8%)

a Patients were allowed to select multiple items to answer this question.

*Although other indicated.

b Other treatment currently received included: "Dexamethasone" (n=3), "Steroids" (n=3), "Zometa" (n=1), "Venetoclax" (n=1), "Cke" (n=1), "Dex, Venclexta" (n=1), "Radiation" (n=1), "Empliciti" (n=1).

c Defined as having received all three of the following treatments: immunomodulatory drug (IMiD), proteasome inhibitor (PI), and anti-CD38.

d Defined as having received at least one of the following treatments: BCMA/CD3 Bispecific Ab, or CAR-T cells.

### 3.2 Patient preference for treatment attributes

The consistency rate for the holdout task was 76.5%, indicating a good level of quality of responses (25, 28).

**Figure 3:**
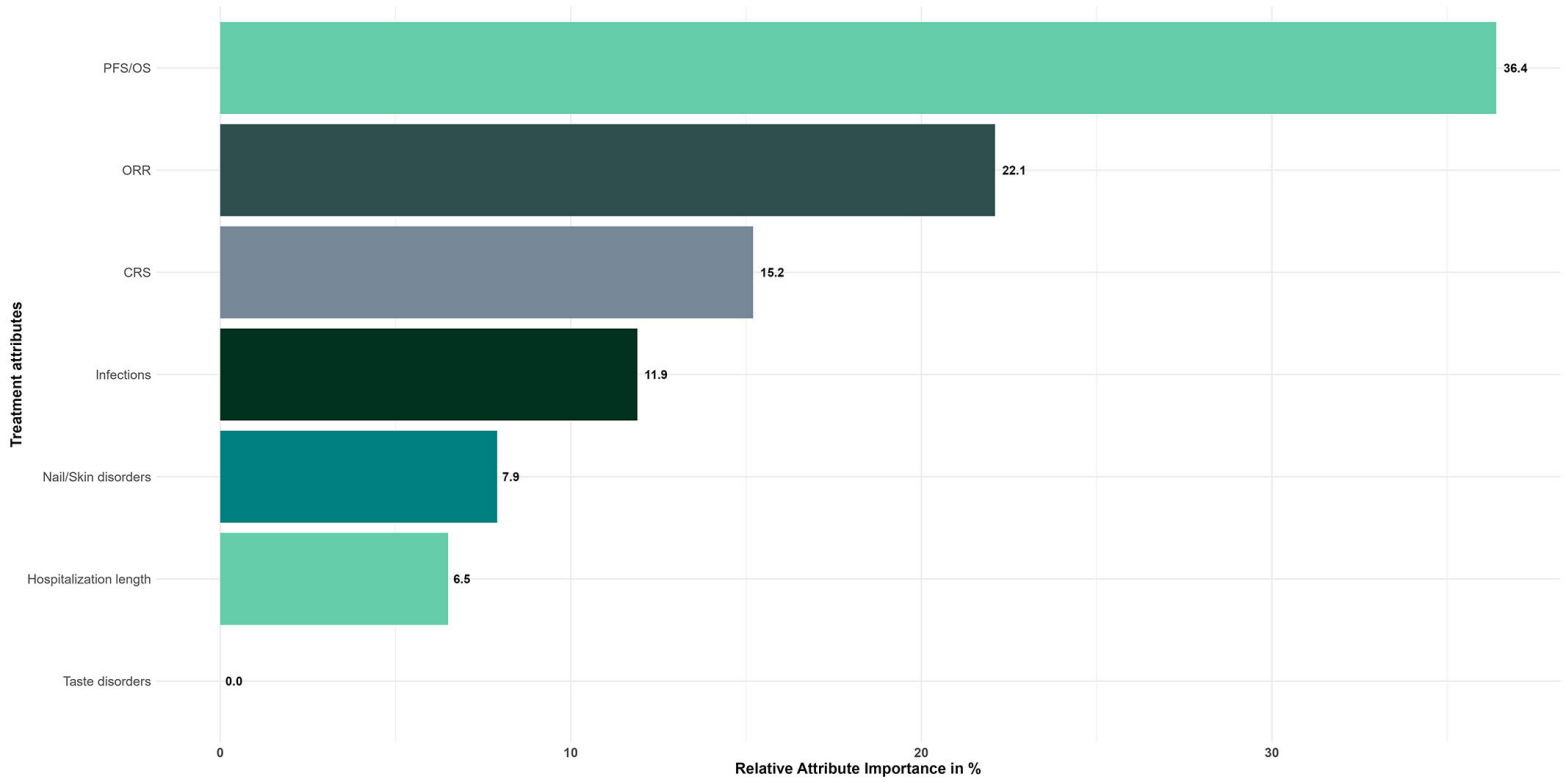
Relative attribute importance scores for treatment attributes - Overall study population (N=149). Reference levels of each attribute were defined as follows: PFS/OS, 4/9 months; ORR, 30%; CRS, 0%; Infections, 50%; Nail/Skin disorders, 0%; Taste disorders, 0%; Hospitalization length, 0 days.

Based on the CL model results, patients ranked the importance of the different attributes for their treatment choice (**Figure 3**). Patients showed a clear preference for attributes related to treatment efficacy, with the combination of PFS and OS ranking first (RAI: 36.4%), followed by ORR (RAI, 22.1%). The two efficacy attributes accounted for more than half of the decision making. With the exception of taste disorders (RAI: 0%), which were considered the least important factor, attributes representing adverse events were ranked third (RAI of CRS, 15.2%), fourth (RAI of infections, 11.9%) and fifth (RAI of nail/skin disorders, 7.9%) in patients' treatment decision-making processes. The length of hospitalization at the start of treatment, which was ranked sixth in terms of importance, was not a significant factor influencing patient preferences (RAI, 6.5%).

**Figure 4:**
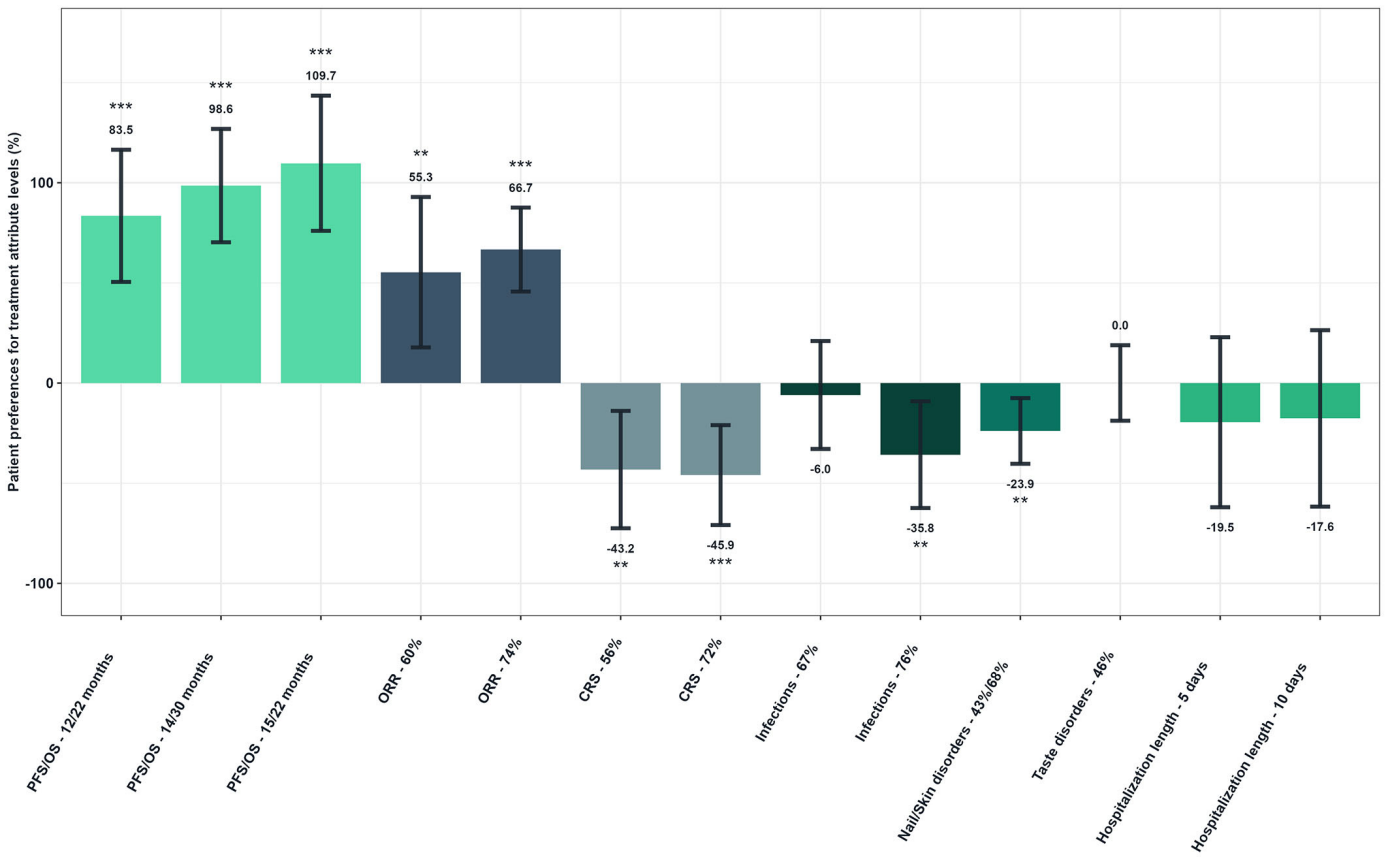
Patient preferences for treatment attribute levels - Overall study population (N=149). Reference levels of each attribute were defined as follows: PFS/OS, 4/9 months; ORR, 30; CRS, 0%; Infections, 50%; Nail/Skin disorders, 0%; Taste disorders, 0%; Hospitalization length, 0 days. As examples to illustrate the interpretation of the results, the probability of choosing a treatment was increased by 83.5% when PFS/OS increased from reference level to 12/22 months. Conversely, it decreased by 43.2% when the risk of CRS was increasing from reference level to 56%.

The probability of selecting a treatment was found to increase significantly (p<0.001) by 109.7% when the PFS and OS were observed to rise from the reference level (4/9 months) to 15 and 22 months, respectively (**Figure 4**). The probability also increased by 66.7% when the ORR rose from the reference level (30%) to 74% (p<0.001). Conversely, the probability of selecting a treatment decreased significantly (p<0.001) by 45.9% when the risk of CRS increased from the reference level (0%) to 72%. It also decreased by 35.8% when the risk of infections increased from the reference level (50%) to 76% (p=0.009), and by 23.9% when the risk of nail and skin disorders increased from the reference level (0%) to 43% and 58% respectively (p=0.004). The probability of choosing a treatment was not significantly reduced when the risk of taste disorders increased, nor when the length of hospital stay was lengthened (p-values>5%).

**Figure 5:**
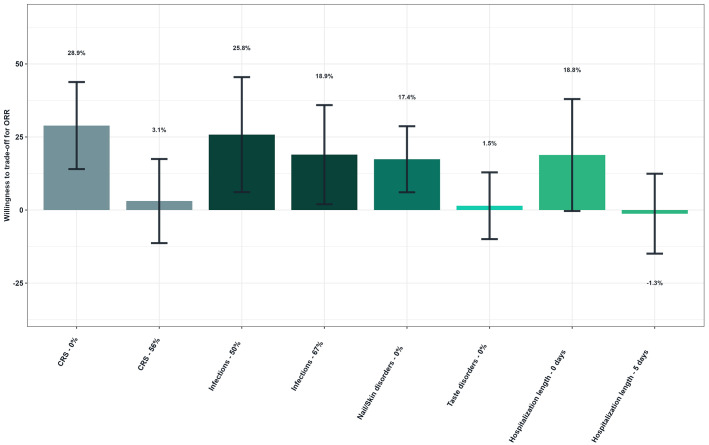
Patients’ willingness to trade off for overall response rate - Overall study population (N=149). Reference levels of each attribute were defined as follows: CRS, 72%; Infections, 76%; Nail/Skin disorders, 43%/68%; Taste disorders, 46%; Hospitalization length, 10 days. As an illustrative example, patients would be willing to accept a higher risk of CRS (72% over no risk) if the hypothetical treatment provided a 28.9% increase in ORR.

**Figure 5** shows patients’ willingness to trade off for ORR (**Figure 5**). Patients would be willing to accept a high risk of CRS (72% over no risk) if the hypothetical treatment provided a 28.9% increase in ORR. Similarly, patients would tolerate a 76% risk of infections (over 50%) in exchange for an additional 25.8% ORR. Patients would also be willing to accept a 43% and 68% risk of nail and skin disorders to gain 17.4% of ORR.

In all subgroup analyses, efficacy attributes were always preferred (**Appendix 2 in the Supplementary Materials**). While no significant differences in patient preferences were observed between earlier and later-line treatment settings, PFS and OS combination was consistently ranked as the top priority. Patients receiving later-line treatments ranked CRS risk as their second priority, whereas those in earlier-line settings prioritized treatments with the highest ORR. Additionally, patients previously exposed to BCMA bispecific antibodies or CAR-T therapies tended to place greater emphasis on ORR, whereas non-exposed patients prioritized PFS/OS. However, the interpretation of these findings is limited by the reduced sample sizes in subgroup analyses, which may preclude the identification of statistically significant differences.

### 3.3 Impact of the disease on life and treatment burden

In terms of the impact of the disease on life and treatment burden, most patients were unable to engage in strenuous activity but were nevertheless capable of undertaking light work (42.3%). A total of 38.9% were ambulatory and capable of self-care, yet unable to carry out any work. Only 12.8% were fully active without restriction. The primary factors influencing the selection of a treatment were the potential long-term impact on health and well-being (58.4%), the recommendations from HCPs (53.0%), and the ability to maintain usual daily activities during treatment (31.5%). The most bothersome consequence of changing MM treatment, as ranked by 28.2% of patients, was the risk of severe side effects, while 11.4% of patients identified adapting to a new form of treatment as their primary concern. Regarding treatment cost, 50% of patients reported that arranging insurance coverage for a new treatment was or would be a bothersome consequence of changing their MM therapy, with 10% identifying it as the most bothersome. Additionally, 24% of patients indicated that insurance coverage or financial considerations influenced their choice of oncologist for MM management. A total of 24.8% of patients indicated that they were not bothered by treatment changes.

## 4 Discussion

As novel therapeutic modalities enhance the prognosis of MM, evaluating their efficacy and safety profiles becomes crucial, particularly given new benefit/risk profile with the emerging therapies. A recent network meta-analysis comparing 34 treatment options for RRMM has demonstrated the complexity of treatment decisions due to the potential toxicities associated with these therapies (29). In addition, another multinational study involving patients with MM revealed concerns about severe side effects, including permanent organ damage, bone fractures, and neuropathic complications. This further emphasizes the importance of considering the balance between toxicity and efficacy when making treatment decisions (30). The significant side effects commonly associated with RRMM treatments, including neuropathy, infections, digestive problems, anemia, CRS, and vision problems, underscore the need for comprehensive patient-provider discussions regarding treatment options. This enables the provision of comprehensive information to patients regarding both severe and milder adverse events that may impact their daily lives and independence.

In 2021, the International Myeloma Working Group (IMWG) updated their treatment guidelines for RRMM, recommending a personalized approach based on patient’s history and treatment responses (31). The intention of these guidelines is to assist healthcare providers in making complex treatment choices. The objective of these recommendations is to facilitate the process of shared decision-making process for both HCPs and patients who are confronted with complex choices. This involves providing patients with comprehensive information about the latest therapeutic options, thereby facilitating their ability to make well-informed decisions regarding their care. Consequently, patients can evaluate the benefits of novel therapies in comparison to the potential adverse effects and other pertinent factors, considering their individual preferences and overall QoL. Therefore, understanding patient preferences is crucial for the optimal treatment decision for RRMM. In parallel, the IMWG emphasizes that healthcare providers consider not only clinical efficacy and safety but also patient-specific factors such as frailty, comorbidities, and treatment goals, highlighting the need for alignment between medical judgment and patient values in shared decision-making.

This study used a DCE to assess the preferences of 149 patients with RRMM to gain insights into the influence of various treatment attributes on their decision-making processes. The results demonstrated that patients assigned the greatest importance to efficacy attributes, particularly PFS and OS, which were rated as the most important (RAI, 36.4%), followed by ORR. Patients were willing to tolerate a higher risk of adverse events such as cytokine release syndrome (CRS) and infections to gain in exchange for an increase in ORR. Notably, the model estimated that patients would accept up to a 72% risk of CRS for a 29% absolute increase in ORR. While this finding reflects the strong preference for efficacy observed across the sample, it should be interpreted with caution. These estimates are derived from a hypothetical, controlled choice experiment and may not fully capture the complexities of real-world decision-making, which occurs under clinical uncertainty and physician guidance. In practice, patients’ actual choices are influenced by factors such as physician recommendations, emotional responses, trust, health literacy, and the way risks and benefits are communicated. Furthermore, although the CRS and ORR levels used in the DCE were grounded in clinical trial data to reflect plausible ranges seen with emerging therapies, individual tolerance for side effects in a real clinical context may be more conservative. Therefore, while these trade-off values provide useful directional insights into patient preferences, they are not intended to predict exact behavior in clinical settings. This perspective also helps contextualize why patients in this study placed relatively less weight on milder, less life-threatening complications, such as taste, skin, and nail disorders, which had a relatively low impact on patient choices, compared to survival outcomes and severe side effects.

Prior studies using DCEs in the context of MM have consistently demonstrated that patients prioritize treatment efficacy while also considering the route of administration, toxicity, survival, remission period, and costs (32, 33). For example, a DCE was conducted in 2022, involving 296 RRMM patients across the USA, United Kingdom (UK), and several European countries. The findings indicated that the most influential attributes in treatment decision-making were a 25 to 85% increase in ORR and a six-month to two-year OS increase, together accounting for approximately 50% of the decision weight. This study revealed that although patients place a high value on treatment efficacy, many are willing to accept side effects such as neuropathy, fatigue, or cognitive impairment in exchange for improved survival rates (32). Similarly, another study reported that patients placed a high valued on increased life expectancy and time to relapse, with pain and fatigue being identified as significant considerations (34). A notable finding is that patients' current health state exerts a greater influence on treatment preferences than their disease status, suggesting that individual health conditions play a pivotal role in decision-making processes. In addition, the findings of Fifer et al.'s research suggested that patients attribute considerable importance to treatment efficacy, particularly in terms of OS, while also taking into account factors such as mode of administration and side effects (35). The findings indicated the necessity of incorporating patient preferences into treatment decision-making for MM and reflects the recurring concern reported by patients about extending survival, despite the potential negative impact of certain adverse effects. Patients demonstrated a propensity to make trade-offs between efficacy, adverse effects, and administration procedures in pursuit of enhanced health outcomes (32).

Although this study primarily evaluated attributes relevant to bispecific therapies, the findings also align with patient preferences observed in studies of CAR T-cell therapy (36, 37), further underscoring the importance of efficacy and the willingness of patients to tolerate certain risks for improved survival outcomes.

This is the first DCE study that included GPRC5D-related treatment attributes. We found that GRPC5D related symptoms such as taste, skin, and nail AEs had relatively low importance in patients’ preference. The lower prioritization of taste, nail, and skin disorders in our study suggests that these issues are either less familiar or less significant to RRMM patients, or they may not be perceived as major barriers to treatment adherence, despite their potential impact on QoL. One plausible explanation is that very few patients in our sample had direct experience with these symptoms —due to the novelty of the GPRC5D target—leading to reduced salience in their preference formation. Indeed, unfamiliarity with these AEs may have limited patients’ ability to fully assess their potential burden, thereby affecting the weight given to these attributes in the DCE. To overcome patients’ unfamiliarity, educational context was provided during the survey, and detailed attribute definitions were given to participants (**Appendix 1, Section B in Supplementary Materials**). Nevertheless, this pattern may also reflect a broader prioritization of survival and treatment efficacy over QoL-related concerns, particularly in the context of advanced disease. This trend is consistent with findings from other studies, such as that published by Thomas et al., which showed that patients with RRMM rather prioritize treatment efficacy and survival outcomes over potential side effects, even when the latter ones may significantly impact their QoL (32).

Moreover, the relatively low awareness of these milder adverse effects may be attributed to a predominant focus on more life-threatening complications, such as CRS and infections, in clinical discussions. Fifer et al. pointed out the tendency for severe adverse events to dominate patient-provider conversations, thereby overshadowing discussions about milder adverse events such as taste changes and skin reactions (35). This highlights the necessity for improvements in the fields of patient education and shared decision making. Although severe complications naturally capture more attention, healthcare providers should also address the importance of less severe adverse events to ensure that patients are fully informed about all potential treatment-related effects.

This study has several limitations. First, the reliance on self-reported data for diagnostic purposes, the assessment of disease severity, and the characterization of clinical features introduce the potential for recall bias or inaccuracies in reporting, as these details were not verified in medical records or by physicians. This lack of objective corroboration may impact the reliability of the findings on patient characteristics. Second, the sampling method may introduce a selection bias. Participants were sourced from the Carenity platform and local partners, which may limit the generalizability of the results. Patients with more advanced disease or severe symptoms may be underrepresented, given that they are often less likely to engage with online surveys. Third, the sample skewed toward highly educated, White patients, which further limits the generalizability of the findings—particularly in the context of MM, a disease that disproportionately affects individuals of African descent. This underrepresentation of racially and ethnically diverse populations, along with the overrepresentation of highly educated respondents, may affect the applicability of the stated preferences observed in this study. These sampling imbalances highlight the need for broader and more inclusive recruitment strategies (e.g., in-clinic or telephone-based approaches) that ensure adequate representation of underserved groups in future preference research.

Additionally, this study focused primarily on attributes associated with bispecific therapies, which may limit generalizability to other treatment modalities in RRMM. The exclusion of cost-related attributes, which frequently influence patient decision-making in real-world settings, suggests that future studies should consider integrating these factors. Furthermore, while this study assessed patient preferences regarding adverse events such as taste, skin, and nail disorders, only 16% of the surveyed patients had received a bispecific antibody, of which only five patients had been treated with talquetamab. Consequently, most respondents may not have had direct experience with these side effects, potentially impacting the accuracy of their risk perceptions and preferences. However, this proportion is consistent with real-world treatment patterns: approximately 45% of patients with ≥4 prior lines of therapy received a BCMA bispecific antibody in 2023 (38) and in our sample, 30% of heavily pretreated patients had received bispecifics. Given the challenges of reaching late-line patients in online surveys, this reflects a reasonably representative distribution within the current clinical landscape.

Additionally, the study is subject to the inherent methodological limitations common to DCEs. Despite an accuracy of 76.5% in this study, it is possible that stated preferences may differ from actual treatment decisions in a real-life context. Desjeux et al. suggest that answers of patients to choice tasks may potentially be different from what they would actually choose if faced with the alternative in real life (39). Moreover, the authors highlight a learning effect of the patients who possibly tend to set their choice according to the first profiles or attributes which are proposed to them. This bias is mitigated by the fact that not all respondents will see the same sequence of attributes. Cognitive fatigue resulting from repeated choice tasks may also impact on the accuracy of responses, thereby complicating the interpretation of results. It is notable that the DCE did not account for other potentially influential factors, such as out-of-pocket costs, deductibles, or the presence of comorbidities. The exclusion of these elements restricts the scope of the findings and suggests that future studies should integrate these factors to provide a more comprehensive understanding of patient preferences.

Finally, emerging molecular insights in RRMM—such as alterations in the MAPK signaling pathway, including BRAF mutations and dysregulation of the Capicua transcriptional repressor—are increasingly relevant to disease progression, drug resistance, and extramedullary disease (40). As precision therapies targeting these molecular features gain clinical traction, patient preferences may evolve accordingly. Incorporating biomarker-driven attributes into future DCEs may be critical for accurately capturing preferences in the context of personalized medicine. Doing so would help align patient-centered care with advances in genomic oncology.

Despite these limitations, the study's key strengths include the rigorous application of DCE methodology, which effectively captures patient preferences by mimicking real-world trade-offs. Additionally, the sample size exceeded the minimum recommended for this type of analysis, enhancing the reliability of the results.

In view of these findings, it is of utmost importance to gain a deeper understanding of patient preferences in the management of RRMM. It would be beneficial for future studies to aim for the incorporation of a more diverse patient population and explore the impact of a broader range of factors influencing treatment decisions. By enhancing our understanding of patient preferences and incorporating additional variables, research can facilitate the development of more personalized treatment strategies that align with the needs and values of patients and optimize treatment decisions.

## Data Availability

The raw data supporting the conclusions of this article will be made available by the authors, without undue reservation.
