# Supplementary material for: A discrete choice experiment analysis to understand patient preferences for multiple myeloma treatments
Source: Front Oncol. 2025 Sep 16;15:1628121. doi: 10.3389/fonc.2025.1628121 (PMC12479298; doi:10.3389/fonc.2025.1628121)
Supplement: Supplementary file 1 [file DataSheet1.pdf]

Supplementary Material

Appendix 1: Survey Questionnaire

A. SCREENER

1. **Your year of birth:** *(YYYY) [Calendar choice]*

   Please select a year in the calendar by clicking the field below.

   [IF <18 YEARS OLD, END OF SURVEY]

2. **Your country of residence:**

   (Single choice)

   Please select only one answer.
   - ☐ United States of America
   - ☐ Other **[END OF SURVEY]**

3. **Have you been diagnosed with multiple myeloma?**

   (Single choice)

   Please select only one answer.
   - ☐ Yes, I am newly diagnosed with multiple myeloma **[END OF SURVEY]**
   - ☐ Yes, I am currently in remission from multiple myeloma (a decrease in or disappearance of signs and symptoms)
   - ☐ My multiple myeloma is currently relapsing, or I have refractory multiple myeloma (persistence or progression of the disease despite previous therapies)
   - ☐ I do not have multiple myeloma **[END OF SURVEY]**

4. **Which healthcare professional (HCP) diagnosed your multiple myeloma?**

   (Single choice)
   Please select only one answer.
   - ☐ General practitioner
   - ☐ Oncologist
   - ☐ Hematologist
   - ☐ Radiologist
   - ☐ Other, please specify: [Free field]
   - ☐ My multiple myeloma was not diagnosed by a HCP **[END OF SURVEY]**

5. **Are you currently receiving treatment for your multiple myeloma?**

   (Single choice)

Please select only one answer.

- ☐ Yes
- ☐ No

First-line treatment or therapy simply refers to the first treatment your doctor has chosen for your multiple myeloma. Second-line treatments are used when the first-line treatment has not improved your multiple myeloma, or if it was effective for some time and then the multiple myeloma progressed. Third-line treatments are used when second-line treatment has not improved your multiple myeloma, or if it was effective for some time and then the multiple myeloma progressed, and so on.

With the following question, we'd like to know how many treatment lines you've received so far.

6. Have you had to change to a different treatment because a treatment line did not work or because you relapsed, and if so, how often?

(Single choice)

Please select only one answer.

- ☐ No, I never had to change to a different treatment **[END OF SURVEY]**
- ☐ Yes, I had to change to a different treatment 1 time
- ☐ Yes, I had to change to a different treatment 2 times
- ☐ Yes, I had to change to a different treatment 3 times
- ☐ Yes, I had to change to a different treatment 4 times
- ☐ Yes, I had to change to a different treatment 5 times or more

B. DISCRETE CHOICE EXPERIMENT (DCE)

For the rest of the survey, you will be asked to imagine you are in a situation where you have to choose between two treatment options with specific characteristics. These treatment options and their characteristics are only hypothetical. This means that these treatments may not apply to you, and that their characteristics cannot be transposed to your personal health situation.

On the following pages, we will present you with 9 pairs of two hypothetical medication options (A vs. B) for the treatment of your Multiple Myeloma. The alternative options will differ by the 8 treatment attributes listed on the left and right. Over the course of the survey, we will ask you to weigh up the treatments shown against each other and decide, based on the attributes, which treatment you would prefer in theory. These 8 attributes can be described as follows:

Time without progression of multiple myeloma – Describes the Time (in months) until your multiple myeloma comes back or starts advancing, or until death.

Lifespan – Describes the Time (in months) until the death.

Likelihood of responding to treatment – Describes the proportion of patients who experience a favorable response involving a reduction in multiple myeloma symptoms, improvement in overall health, and/or a decrease in the progression of the disease.

Immune storm (also known as CRS) – Describes the proportion of treated patients who experience a mild to moderate excessive immune system response, resulting in symptoms such as fever, chills, low blood pressure, breathing difficulties, and body aches, during the treatment.

Infections – Describes the proportion of treated patients who experience a weakening of the immune system and an increased vulnerability to acquiring infections, some of which may be severe and necessitate hospitalization, during the treatment.

Skin and/or nail disorders – Describes the proportion of patients who experience adverse events related to skin and/or nail disorders, characterized by unwanted changes to the skin and/or nails due to the medication being taken. These events may manifest as rashes, itching, peeling, redness, discoloration of the skin, and changes in the appearance of nails.

Taste disorder – Describes the proportion of treated patients who experience a taste disorder (can range from lack of taste to loss of taste) during the treatment.

Hospitalization to start treatment – Describes the duration of hospitalization at the start of treatment to ensure that the patient is monitored and tolerates the treatment.

Scenario example n°1

7. **Please review the following treatment options and choose the treatment option you would prefer in the current context of your multiple myeloma**

You can find the full attribute descriptions here.

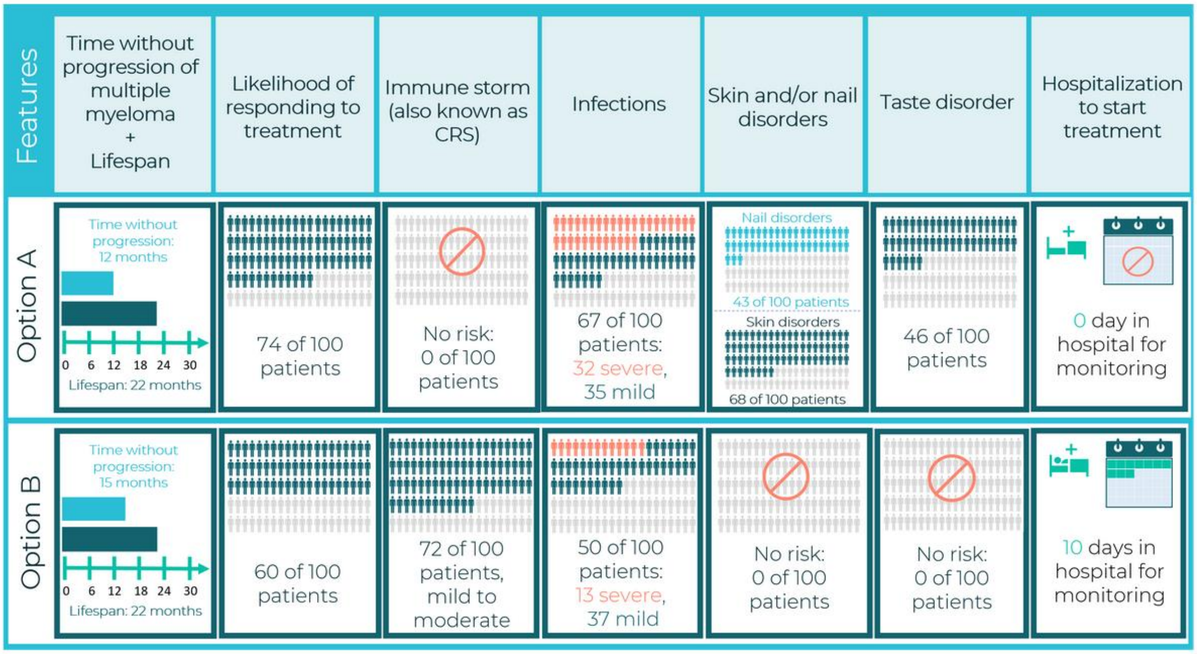

☐ Option A

- ☐ Option B

Scenarios n°2 to 9

15. Please review the following treatment options and choose the treatment option you would prefer in the current context of your multiple myeloma

You can find the full attribute descriptions here.

C. SOCIODEMOGRAPHIC AND MEDICAL PROFILE

Instructions

Thank you for answering these questions related to your treatment preferences.

In this section, you will be asked a few more questions related to your demographic profile, your multiple myeloma and the medication(s) you are receiving for your multiple myeloma.

16. You are:

(Single choice)

Please select only one answer.

- ☐ Male
- ☐ Female
- ☐ Transgender
- ☐ Non-binary
- ☐ Prefer to self-describe *Please specify:* [Free field]
- ☐ Prefer not to answer

17. Which races, ethnicities or origins describe you?

(Multiple choice)

Please select all that apply.

- ☐ Hispanic and/or of Latin origin
- ☐ Black and/or of African origin
- ☐ White/Caucasian and/or of European origin
- ☐ Middle Eastern and/or of Northern African origin
- ☐ Native Hawaiian and/or of Pacific Islander origin
- ☐ Far East or Southeast Asian origin
- ☐ Asian Indian origin
- ☐ Native American, Alaskan Native and/or Indigenous origin
- ☐ Prefer to self-describe, I am: *[Please specify]* **[EXCLUSIVE]**
- ☐ Prefer not to share or don’t know **[EXCLUSIVE]**

18. What is the highest educational or equivalent work-related qualification that you have?

(Single choice)

Please select only one answer.
- ☐ I did not finish high school
- ☐ High school diploma
- ☐ 2-year college degree
- ☐ Bachelor's degree
- ☐ Master's degree
- ☐ PhD (doctorate)
- ☐ Other *[Please specify]*

19. Where do you consider you currently live?

(Single choice)

Please select only one answer.
- ☐ In a urban area
- ☐ In suburban area
- ☐ In a rural area

20. Which of the following best describes your personal income last year?

(Single choice)

Please select only one answer.
- ☐ $0-$24,999
- ☐ $25,000-$49,999
- ☐ $50,000-$74,999
- ☐ $75,000-$124,999
- ☐ $125,000-$199,999
- ☐ $200,000+
- ☐ Prefer not to answer

21. When was your multiple myeloma diagnosed?

(If you do not know the exact year and month, please give us an estimate)

[Choice on the calendar] (mm/yyyy)
- ☐ I don't know

[Q22 is only asked for patients with Q5='Yes']

22. What treatments are you <u>currently</u> taking for your multiple myeloma?

(Multiple choice)

Please select all that apply.
- ☐ Selinexor (Xpovio®)
- ☐ Proteasome inhibitors (e.g. bortezomib (Velcade®), ixazomib (Ninlaro®), carfilzomib (Kyprolis®))
- ☐ Immunomodulatory drug (e.g. lenalidomide (Revlimid®), pomalidomide (Pomalyst®, Imnovid®))
- ☐ Monoclonal antibody anti-CD38 (e.g. daratumumab (Darzalex®), isatuximab (Sarclisa®))
- ☐ BCMA/CD3 Bispecific Ab (e.g., teclistamab (Tecvayli®), elranatamab (Elrexfio®))
- ☐ GPRC5D/CD3 Bispecific Ab (e.g., talquetamab (Talvey®))

- ☐ CAR-T cells (e.g. cilta-cel (Carvykti®), idecabtagene vicleucel (Abecma®))
- ☐ Stem cell transplant
- ☐ Other *[Please specify]*
- ☐ I don't know **[EXCLUSIVE]**

23. What treatments have you taken in the past for your multiple myeloma?

(Multiple choice)

Please select all that apply.

- ☐ Selinexor (Xpovio®)
- ☐ Proteasome inhibitors (e.g. bortezomib (Velcade®), ixazomib (Ninlaro®), carfilzomib (Kyprolis®))
- ☐ Immunomodulatory drug (e.g. lenalidomide (Revlimid®), pomalidomide (Pomalyst®, Imnovid®))
- ☐ Monoclonal antibody anti-CD38 (e.g. daratumumab (Darzalex®), isatuximab (Sarclisa®))
- ☐ BCMA/CD3 Bispecific Ab (e.g., teclistamab (Tecvayli®), elranatamab (Elrexfio®))
- ☐ GPRC5D/CD3 Bispecific Ab (e.g., talquetamab (Talvey®))
- ☐ CAR-T cells (e.g. cilta-cel (Carvykti®), idecabtagene vicleucel (Abecma®))
- ☐ Stem cell transplant
- ☐ Other *[Please specify]*
- ☐ I don't know **[EXCLUSIVE]**

24. Have you ever been hospitalized for several days because of your multiple myeloma?

(Single choice)

Please select only one answer.

- ☐ Yes à *Please specify how many days (Numerical field, minimum=2)*
- ☐ No

25. Which type of insurance are you using to cover your multiple myeloma treatment?

(Single choice)

Please select only one answer.

- ☐ State/Public insurance
- ☐ Private insurance
- ☐ Combination of private and State/Public Insurance
- ☐ Other *[Please Specify]*
- ☐ I don't know **[EXCLUSIVE]**

26. Which of the following conditions are you currently living with outside of your multiple myeloma?

(Multiple choice)

Please select all that apply.

- ☐ Cardiovascular disease (e.g. high blood pressure, heart attack, heart failure, stroke)
- ☐ Metabolic diseases (e.g., diabetes, obesity)
- ☐ Pulmonary disease (e.g. pneumonia, asthma)

- ☐ Renal disease (e.g. kidney failure)
- ☐ Mood disorders (e.g. depression, bipolar disorder)
- ☐ Autoimmune disease(s) (e.g. lupus, psoriatic arthritis, psoriasis, thyroid disease)
- ☐ Inflammatory bowel disease (e.g. Crohn's disease, ulcerative colitis)
- ☐ Rheumatic diseases (e.g. polyarthritis, spondylarthritis)
- ☐ Bone and skeletal disorders
- ☐ Cancer *[Please specify]*
- ☐ Other *[Please specify]*
- ☐ None **[EXCLUSIVE]**

D. IMPACT OF THE DISEASE ON LIFE & TREATMENT BURDEN

Instructions

Thank you for answering these questions related to sociodemographic and medical profile.

This section of the survey includes questions concerning the impact of your multiple myeloma on your quality of life.

27. Regarding your activity and physical function, how would you generally rate your activity over the past month?

(Single choice)

Please select only one answer.

- ☐ Fully active, able to carry on all pre-disease performance without restriction
- ☐ Restricted in physically strenuous activity but ambulatory and able to carry out work of a light or sedentary nature, e.g., light house work, office work
- ☐ Ambulatory and capable of all self-care but unable to carry out any work activities. Up and about more than 50% of waking hours
- ☐ Capable of only limited selfcare, confined to bed or chair more than 50% of waking hours
- ☐ Completely disabled. Cannot perform any selfcare. Totally confined to bed or chair

[Q28a is only asked for patients with Q5='Yes']

28. a. Which consequences of changing your multiple myeloma treatment would you consider the most bothersome?

The first item you select is the most bothersome, and so on. You can change the order or remove an item by clicking on it again. It's not necessary to sort all items, just the ones that make sense to you.

1= this item is the most bothersome, 2= this item is the second most bothersome, etc.

(Ranking question, randomized items)

- ☐ Adapting to a new frequency of medication intake
- ☐ Being hospitalized for a few days at the start of treatment to ensure that it is well tolerated
- ☐ Risking severe side effects (severe adverse effects or events requiring hospitalization)

- ☐ Risking mild side effects (adverse effects or events that can be treated or tolerated in an outpatient care setting)
- ☐ Adapting to a new form of treatment (e.g. pills of different size, different number of pills, starting injectable treatments)
- ☐ Arranging the coverage of my new treatment with my health insurance
- ☐ Visiting my doctor more frequently in the following months
- ☐ Changing treatment does not bother me **[EXCLUSIVE]**

[Q28b is only asked for patients with Q5=’No’]

28. b. Referring to any previous treatments you may have had, which consequences of changing your multiple myeloma treatment did you consider the most bothersome?

The first item you select is the most bothersome, and so on. You can change the order or remove an item by clicking on it again. It's not necessary to sort all items, just the ones that make sense to you.

1= this item is the most bothersome, 2= this item is the second most bothersome, etc.

(Ranking question, randomized items)

- ☐ Adapting to a new frequency of medication intake
- ☐ Being hospitalized for a few days at the start of treatment to ensure that it is well tolerated
- ☐ Risking severe side effects (severe adverse effects or events requiring hospitalization)
- ☐ Risking mild side effects (adverse effects or events that can be treated or tolerated in an outpatient care setting)
- ☐ Adapting to a new form of treatment (e.g. pills of different size, different number of pills, starting injectable treatments)
- ☐ Arranging the coverage of my new treatment with my health insurance
- ☐ Visiting my doctor more frequently in the following months
- ☐ Changing treatment did not bother me **[EXCLUSIVE]**

29. Which of the following factors influenced your decision when choosing an oncologist for your multiple myeloma management?

(Multiple choice)

Please select all that apply.

- ☐ Recommended by a healthcare professional
- ☐ Recommended by friends or family
- ☐ Specific expertise in Multiple Myeloma
- ☐ Proximity or convenience of the clinic/hospital
- ☐ Reputation of the treatment center/hospital
- ☐ Insurance coverage or financial considerations
- ☐ Research and online reviews
- ☐ Availability of clinical trials or experimental treatments
- ☐ Positive patient experiences or testimonials
- ☐ Other *[Please specify]*
- ☐ I didn't choose the oncologist treating my multiple myeloma **[EXCLUSIVE]**

30. Beyond the general treatment characteristics (efficacy, mode of administration, and side effects), which of the following aspects influenced your decision in choosing a treatment for your multiple myeloma?

(Multiple choice)

Please select all that apply.

- ☐ Impact on long-term health and well-being
- ☐ Recommendations from support groups or patient communities
- ☐ Recommendations from healthcare professionals
- ☐ Insights from other patients' experiences
- ☐ Potential impact on emotional and mental well-being
- ☐ Alignment with personal treatment preferences or values
- ☐ Convenience of appointment scheduling and treatment process
- ☐ Ability to continue usual daily activities during treatment
- ☐ Suitability for your age and lifestyle
- ☐ Potential impact on social interactions and relationships
- ☐ Level of involvement required from caregivers or family members
- ☐ Information from reputable online sources
- ☐ Compatibility with dietary or lifestyle choices
- ☐ Access to additional support services (psychological, nutritional, etc.)
- ☐ Potential impact on fertility or family planning
- ☐ Consideration of travel requirements or distance to treatment center
- ☐ Other *[Please specify]*
- ☐ I didn't choose my multiple myeloma treatment **[EXCLUSIVE]**

31. During your care pathway, do you feel that your medical team has communicated clearly with you about your treatment options?

(Single choice)

- ☐ Yes
- ☐ No **à 29b**

31b. Why?

(Please be specific in your answer and provide as many details as possible)

Open field text

Appendix 2: Summary of RAI of treatment attributes per subgroup

Treatment Attributes	PFS/OS	ORR	CRS	Infections	Nail/Skin disorders	Taste disorders	Hospitalization length
Overall (n=149)	36.4%	22.1%	15.2%	11.9%	7.9%	0%	6.5%
1 or 2 prior lines of treatment (n=98)	36.8%	22.4%	10.4%	13.2%	7.1%	2.2%	7.9%
3 or more prior lines of treatment (n=51)	30.9%	20.8%	23.4%	11.9%	6.9%	2.7%	3.5%
4 or more prior lines of treatment (n=28)	34.8%	19.1%	19.9%	11.4%	8.7%	1.4%	4.9%
Age below 65 (n=85)	29.7%	20.4%	8.4%	11.2%	9.3%	0%	21%
Age above 65 (n=64)	36.4%	22.5%	18.8%	6%	1.4%	1.2%	13.7%
Female (n=76)	34.7%	21%	14%	7.6%	8.4%	2.6%	11.6%
Male (n=72)	34.1%	20.7%	17.8%	15.8%	7.1%	3%	1.4%
Disease duration below 5 years (n=70)	34.8%	26.3%	10.4%	15.3%	5.2%	7.5%	0.4%
Disease duration above 5 years (n=73)	30.1%	21.2%	19.7%	9%	8.8%	3.7%	7.4%
Urban area (n=68)	19.3%	32.3%	11.1%	12.6%	7.4%	3.6%	13.6%
Suburban area (n=61)	46.8%	11.4%	14.6%	10.5%	8.8%	2.5%	5.4%
Income <$50,000 (n=48)	32.6%	16.4%	23.6%	9.1%	6.9%	4.5%	6.9%
Income $50,000-$74,999 (n=39)	27.5%	44.8%	9.1%	8.3%	0.8%	5%	4.5%
Income $75,000+ (n=50)	37.8%	13.4%	9.8%	11.2%	11%	0.7%	16.1%
Fully active or Restricted (n=82)	44.6%	7.5%	10.4%	12.1%	8.2%	4.2%	12.8%

Treatment Attributes	PFS/OS	ORR	CRS	Infections	Nail/Skin disorders	Taste disorders	Hospitalization length
Ambulatory and limited self-care (n=67)	15.7%	38.8%	17.3%	10.1%	4.7%	7.8%	5.7%
Triple-class exposed (n=87)	33.4%	29.5%	15.2%	12.1%	3.1%	4.3%	2.5%
Triple-class not exposed (n=62)	35.9%	9.9%	14.3%	10%	11%	4.7%	14.3%
BCMA bispecific antibody or CAR-T exposed (n=45)	19.8%	37.4%	17.6%	6.6%	9.7%	6.2%	2.7%
BCMA bispecific antibody or CAR-T not exposed (n=104)	40.7%	13.3%	13.4%	12.9%	6.8%	2.5%	10.3%
